# Self-regulated learning and engagement as serial mediators between AI-driven adaptive learning platform characteristics and educational quality: a psychological mechanism analysis

**DOI:** 10.3389/fpsyg.2025.1646469

**Published:** 2025-10-20

**Authors:** Zipei Ouyang

**Affiliations:** School of Marxism, Quzhou College of Technology, Quzhou, ZheJiang, China

**Keywords:** adaptive learning platforms, self-regulated learning, learning engagement, educational psychology, serial mediation, AI in education

## Abstract

The rapid integration of artificial intelligence in educational technology has transformed learning environments, yet the psychological mechanisms through which AI-driven adaptive learning platforms are associated with enhanced educational outcomes remain insufficiently understood. This study investigates the complex mediational pathways linking adaptive learning platform characteristics to educational quality enhancement, examining the sequential relationships between self-regulated learning and learning engagement from a psychological perspective. Drawing on cognitive and motivational theories of learning, we hypothesized that platform features would be associated with educational quality both directly and through the serial mediation of self-regulated learning and engagement. Employing structural equation modeling with data from 625 learners using AI-driven adaptive learning platforms (including Knewton, ALEKS, and Squirrel AI), this research reveals both significant direct relationships between platform characteristics and educational quality and substantial indirect effects through serial mediation. The findings demonstrate that platform features show strong associations with self-regulated learning, which sequentially relates to learning engagement and educational quality. These results advance our understanding of the cognitive and motivational processes through which technological affordances are associated with enhanced learning outcomes. The study provides important insights into the psychological mechanisms underlying effective digital learning, offering evidence-based guidance for designing adaptive learning environments that are related to learner autonomy and engagement. This research contributes to educational psychology by elucidating how AI-driven personalization features correlate with fundamental psychological processes associated with successful learning experiences.

## 1 Introduction

The psychological study of learning in technology-enhanced environments has emerged as a critical domain for understanding how cognitive and motivational processes adapt to and are influenced by digital educational contexts. Contemporary educational psychology faces fundamental questions about the mechanisms through which technological affordances interact with established psychological constructs such as self-regulated learning and engagement to influence educational outcomes. This investigation addresses these questions by examining the serial mediational pathways linking adaptive learning platform characteristics to educational quality through the sequential interaction of self-regulated learning and learning engagement.

### 1.1 Psychological foundations and research context

The integration of artificial intelligence in educational environments presents unique opportunities for psychological research, particularly in understanding how external technological scaffolds interact with internal self-regulatory processes. From a psychological perspective, this technological integration fundamentally alters the cognitive demands placed on learners and the motivational dynamics of the learning process. Self-regulated learning, conceptualized through Zimmerman’s (2002) cyclical model encompassing forethought, performance, and self-reflection phases, represents a critical psychological construct for understanding how learners navigate technologically mediated environments.

The psychological significance of adaptive learning technologies extends beyond their pedagogical applications to encompass fundamental questions about human learning processes. These platforms, exemplified by systems such as Knewton, ALEKS, and Squirrel AI, represent sophisticated applications of psychological principles in technological contexts, offering unprecedented opportunities to examine how external scaffolding influences internal psychological processes. The effectiveness of such intelligent tutoring systems has been documented extensively (VanLehn, 2011; Kulik and Fletcher, 2016), yet the specific psychological mechanisms underlying their effectiveness remain insufficiently understood.

Contemporary psychological research has begun to explore how technological features support self-regulated learning processes (Wong et al., 2019; Guan et al., 2024), yet significant gaps persist in understanding the sequential relationships between technological characteristics, self-regulatory processes, and engagement patterns. While studies have demonstrated the effectiveness of adaptive learning systems (Wang et al., 2023), the specific psychological pathways through which these systems enhance educational outcomes require systematic investigation. This gap is particularly evident in understanding how self-regulated learning and engagement interact as mediating psychological mechanisms in technology-enhanced learning environments.

### 1.2 Theoretical gaps and research imperatives

Despite substantial advances in both cognitive psychology and educational technology, several critical theoretical gaps persist in our understanding of psychological mechanisms in adaptive learning environments. First, existing theoretical frameworks inadequately explain the psychological processes through which technological characteristics influence learning outcomes. While studies have documented various platform features (Guan et al., 2024), the psychological pathways linking these features to enhanced educational quality remain insufficiently conceptualized, particularly regarding the mediating roles of cognitive and motivational processes.

Second, methodological limitations have hindered comprehensive understanding of psychological mechanisms in technology-enhanced learning. Current evaluation approaches often fail to capture the complex, multi-layered nature of psychological processes in educational technology contexts (Sales and Pane, 2020). The epistemological challenges in evaluating computer-based learning environments (Brust et al., 2007) underscore the need for more sophisticated psychological frameworks that can capture the dynamic interaction between technological affordances and psychological processes.

Third, the potential serial mediation effects of psychological constructs in technology-enhanced learning remain theoretically underexplored. While recent research has examined isolated aspects of self-regulated learning in digital environments (Guan et al., 2024), the sequential interaction between multiple psychological mediating mechanisms requires systematic investigation. This theoretical gap is particularly significant given emerging evidence suggesting that technological effectiveness operates through interconnected psychological pathways.

### 1.3 Research purpose and psychological significance

This study addresses these theoretical and methodological gaps by proposing and empirically testing a comprehensive psychological framework for understanding how adaptive learning platforms influence educational quality through specific cognitive and motivational mechanisms. Specifically, we examine the serial mediating roles of self-regulated learning and learning engagement in the relationship between platform characteristics and educational outcomes, grounded in established psychological theories of learning and motivation.

The investigation makes several significant theoretical contributions to educational psychology. First, it advances self-regulated learning theory by examining how technological affordances scaffold and support self-regulatory processes in adaptive learning environments. Second, it extends learning engagement theory by identifying how technological characteristics influence different dimensions of engagement through self-regulatory pathways. Third, it contributes to our understanding of psychological mediation by demonstrating how cognitive and motivational constructs interact sequentially to influence educational outcomes in technology-enhanced contexts.

Methodologically, this study demonstrates the application of sophisticated psychological research techniques for examining complex mediational relationships in educational technology contexts. By addressing the methodological challenges identified by Sales and Pane (2020) and incorporating psychological perspectives on technology-enhanced learning, we advance empirical approaches to understanding psychological mechanisms in digital learning environments.

### 1.4 Sustainable education framework and conceptualization

Sustainable education represents a multidimensional approach to educational development that integrates resource optimization, long-term educational effectiveness, and equitable access principles within technologically enhanced learning environments. Drawing from Harvey and Green’s (1993) multifaceted quality framework, sustainable education encompasses excellence in learning outcomes, fitness for purpose in meeting diverse learner needs, and transformative potential for long-term educational improvement while optimizing resource utilization. This conceptualization extends beyond traditional educational quality metrics to incorporate environmental, economic, and social sustainability dimensions that ensure educational interventions can be maintained and scaled effectively across diverse contexts.

For the purposes of this study, sustainable education is operationalized through three measurable dimensions with specific indicators. Resource efficiency refers to the optimization of educational inputs while maintaining or improving learning outcomes, measured by reductions in per-student instructional time (target: 20–30% decrease) and instructor workload while preserving educational quality indicators. Scalability encompasses the capacity for educational interventions to serve larger populations without proportional increases in support resources, measured by student-to-faculty ratios (target: 500 + students per additional faculty support unit) and platform adoption rates across diverse institutional contexts. Long-term effectiveness represents the persistence of educational benefits beyond immediate intervention periods, measured by learning retention rates at 6-month and 12-month post-intervention assessments and continued platform usage patterns (VanLehn, 2011).

Contemporary digital pedagogy research has advanced understanding of sustainable education through frameworks emphasizing learner-centered approaches that optimize both immediate learning outcomes and long-term resource efficiency (Huang et al., 2024). These frameworks demonstrate how technological innovation can support sustainable educational transformation by simultaneously enhancing pedagogical effectiveness and institutional efficiency. Sustainable education in technology-enhanced contexts specifically refers to educational approaches that maintain high-quality learning outcomes while optimizing resource allocation, ensuring equitable access, and supporting scalable implementation across diverse educational settings.

Within the context of adaptive learning platforms, sustainable education operationalizes through psychological mechanisms that enhance both learning effectiveness and resource efficiency. The integration of self-regulated learning and engagement processes represents a sustainable approach to educational improvement because these psychological capabilities, once developed, continue to benefit learners across diverse learning contexts while reducing the need for intensive instructional support. This aligns with Biggs (2001) constructive alignment principles and Sallis (2014) quality management frameworks, suggesting that sustainable educational approaches prioritize the development of transferable psychological competencies that support lifelong learning while optimizing institutional resource allocation.

## 2 Literature review

### 2.1 Theoretical foundations of self-regulated learning in digital contexts

Self-regulated learning theory encompasses learner-directed psychological processes involving metacognitive, motivational, and behavioral dimensions of learning regulation. Boekaerts’s (1999) foundational work established that self-regulated learning encompasses both cognitive and motivational components, emphasizing the dynamic interaction between learning strategies and motivational beliefs in determining educational outcomes.

Zimmerman’s (2002) cyclical model conceptualizes self-regulated learning as an interactive psychological process encompassing forethought, performance, and self-reflection phases, emphasizing the cyclical nature of learning regulation. This framework has been enhanced by metacognitive perspectives emphasizing task definition, goal setting, strategy enactment, and adaptation in learning processes (Winne and Hadwin, 1998). Pintrich’s (2004) comprehensive framework elaborated the psychological domains of self-regulation, encompassing cognitive, motivational, behavioral, and contextual dimensions of learning regulation.

Recent theoretical developments have extended this understanding by examining the social dimensions of self-regulation, including co-regulation and socially shared regulation in learning environments (Hadwin et al., 2011; Paris and Paris, 2001; Järvelä and Hadwin, 2013). The theoretical sophistication has been advanced through Schunk and Zimmerman’s (1998) examination of how learners develop metacognitive capabilities, enriched by frameworks explicating dynamic interactions between metacognition, affect, and motivation (Efklides, 2011).

In technology-enhanced environments, self-regulated learning extends beyond traditional conceptualizations to encompass how external technological scaffolds interact with internal self-regulatory processes. Research demonstrates how digital platforms support metacognitive development through algorithmic scaffolding mechanisms. Zhang and Hew (2024) demonstrated how semi-supervised recommender systems foster self-regulated learning through personalized content recommendations, while Liu et al. (2017) revealed significant effects of adaptive technologies on metacognitive awareness and strategic planning behaviors. Chen et al. (2023) explored adaptive learning path navigation through knowledge tracing and reinforcement learning algorithms, demonstrating how artificial intelligence can support optimal learning sequences while enhancing learner autonomy.

### 2.2 Learning engagement theory and psychological mechanisms

Learning engagement theory has evolved from behavioral measures to sophisticated multidimensional psychological constructs encompassing behavioral, emotional, and cognitive dimensions of student involvement (Fredricks et al., 2004). Contemporary sociocultural frameworks have enriched this understanding by incorporating structural influences, psychosocial factors, and both proximal and distal educational outcomes, reflecting the complex psychological nature of student engagement in modern educational contexts (Kahu, 2013).

Reschly and Christenson (2012) advanced theoretical understanding by addressing conceptual challenges in engagement research, demonstrating how different theoretical perspectives contribute to nuanced understanding of engagement as a malleable psychological state. Their work reveals the importance of distinguishing between engagement as a behavioral outcome and engagement as an underlying psychological process, emphasizing the need for sophisticated measurement approaches that capture the multifaceted nature of student involvement.

Theoretical developments have emphasized the dynamic psychological nature of engagement, conceptualizing it as a malleable psychological state influenced by both individual and contextual factors (Sinatra et al., 2015). Finn and Zimmer (2012) demonstrated how engagement operates as both psychological state and behavioral indicator, revealing complex relationships between internal motivational processes and observable learning behaviors. This perspective aligns with motivational theories emphasizing the importance of autonomy, competence, and relatedness in fostering psychological engagement (Reeve, 2012).

In technology-enhanced learning environments, research has revealed how technological features influence engagement patterns through enhanced interactivity, personalized learning experiences, and real-time feedback systems that address fundamental psychological needs for competence and autonomy (Krause and Coates, 2008; Trowler, 2010; Zepke and Leach, 2010). The mediating function of engagement has been demonstrated through various psychological pathways, including increased psychological investment, enhanced learning persistence, and improved affective responses to learning tasks (Sinatra et al., 2015).

### 2.3 Adaptive learning technologies and educational quality frameworks

Contemporary adaptive learning platforms represent sophisticated technological implementations of psychological principles, offering unique contexts for examining how external scaffolds influence internal psychological processes. Sachete et al. (2024) developed AdaptiveGPT frameworks demonstrating how artificial intelligence systematically supports learning through intelligent content adaptation and personalized feedback mechanisms. St-Hilaire et al. (2021) conducted comprehensive comparative studies of learning outcomes across different online learning platforms, demonstrating significant variation in effectiveness based on technological design characteristics and psychological support mechanisms.

The conceptualization of educational quality in technology-enhanced learning environments requires sophisticated theoretical frameworks that capture the multifaceted nature of learning outcomes. Harvey and Green’s (1993) foundational work established multiple perspectives on quality in higher education, including excellence, perfection, fitness for purpose, value for money, and transformative potential. Biggs (2001) advanced theoretical understanding through the concept of constructive alignment in quality assurance, emphasizing the critical importance of alignment between learning objectives, teaching methods, and assessment practices in technology-enhanced educational settings.

Contemporary research has extended quality frameworks through multifaceted approaches that incorporate both process and outcome indicators. Tam (2001) demonstrated how quality measurement in technology-enhanced learning requires comprehensive frameworks that capture both immediate learning outcomes and long-term educational benefits. Schindler et al. (2015) contributed through systematic synthesis of quality definitions, while Owlia and Aspinwall (1996) provided frameworks encompassing both educational effectiveness and institutional efficiency. Sallis (2014) demonstrated comprehensive approaches to quality management integrating technological innovation with educational excellence, while Becket and Brookes (2006) examined quality management in university departments. Srikanthan and Dalrymple (2003) developed alternative perspectives for quality assessment emphasizing stakeholder engagement and institutional effectiveness in technology-enhanced contexts.

### 2.4 Research synthesis and hypothesis development

The synthesis of existing psychological literature reveals critical insights regarding the mechanisms through which technological platforms influence educational outcomes while identifying significant theoretical gaps requiring further investigation. The psychological framework emerging from this review suggests a sequential process whereby technological characteristics enhance self-regulatory capabilities, which subsequently facilitate deeper learning engagement and improved educational quality.

Drawing upon this integrated psychological foundation, we propose a comprehensive research framework examining the relationships between adaptive learning platform characteristics, psychological processes, and educational outcomes. Self-regulated learning theory provides the foundational premise for understanding how technological integration supports metacognitive and motivational processes essential for effective learning (Zimmerman, 2002). This theoretical perspective suggests that technological characteristics serve as external scaffolds that support internal psychological processes critical for learning success.

Concurrently, engagement theory illuminates the motivational mechanisms through which technological affordances manifest in educational contexts, particularly through adaptive capabilities that address fundamental psychological needs for competence, autonomy, and optimal challenge (Fredricks et al., 2004). The integration of these theoretical perspectives provides a sophisticated psychological lens for examining how technological affordances support learning through enhanced metacognition, motivation, and engagement.

This theoretical synthesis leads to the development of research hypotheses examining both direct psychological effects and complex mediational pathways:

*H1*: Adaptive learning platform characteristics demonstrate significant positive effects on educational quality indicators.

*H2*: Self-regulated learning mediates the relationship between platform characteristics and educational quality enhancement.

*H3*: Learning engagement mediates the relationship between platform characteristics and educational quality enhancement.

*H4*: Self-regulated learning and learning engagement form a serial mediation chain in the relationship between platform characteristics and educational quality enhancement.

These hypotheses collectively address critical gaps in current psychological understanding, particularly regarding the complex pathways through which technological affordances translate into enhanced educational outcomes through specific psychological mechanisms. By examining both direct effects and mediated psychological pathways, this research framework provides a comprehensive approach to understanding the multifaceted psychological relationships between adaptive learning technologies and educational effectiveness.

## 3 Research design and methodology

### 3.1 Theoretical framework and research design

This investigation employs a quantitative cross-sectional design integrating self-regulated learning theory (Zimmerman, 2002), multidimensional engagement theory (Fredricks et al., 2004), and cognitive scaffolding principles (Winne and Hadwin, 1998) to examine psychological mediation pathways in technology-enhanced learning environments. The theoretical synthesis positions adaptive learning platform characteristics as exogenous variables influencing educational quality through both direct technological effects and complex psychological mediation via self-regulated learning and engagement constructs.

Zimmerman’s (2002) cyclical model of self-regulated learning provides the primary theoretical lens, encompassing forethought, performance, and self-reflection phases that interact systematically with technological affordances. This framework is augmented by Pintrich’s (2004) multidimensional conceptualization incorporating cognitive, motivational, behavioral, and contextual domains of learning regulation. The integration with Fredricks et al.’s (2004) tripartite engagement model—encompassing behavioral, emotional, and cognitive dimensions—establishes a comprehensive psychological framework for understanding technology-cognition interactions in educational contexts.

The research context encompasses higher education institutions implementing adaptive learning platforms (Knewton, ALEKS, Squirrel AI) for minimum one academic year duration. Sample size determination followed structural equation modeling protocols incorporating Cohen’s (1988) power analysis and Monte Carlo simulation procedures (Muthén and Muthén, 2002), establishing minimum requirements for detecting complex mediational relationships with statistical power of 0.90 at *α* = 0.05. A stratified random sampling approach ensured representativeness across institutional characteristics, academic disciplines, and technological implementation levels while addressing potential selection bias and technological proficiency confounds.

### 3.2 Measurement development and psychometric properties

The operationalization of psychological constructs followed rigorous psychometric principles emphasizing theoretical grounding and comprehensive validation procedures. Adaptive learning platform characteristics were assessed through a multidimensional framework encompassing technological affordances that support psychological processes, specifically platform intelligence, personalization capabilities, feedback mechanisms, content adaptation, and interface design as theoretically relevant dimensions supporting metacognitive and motivational functioning.

Self-regulated learning was operationalized through Pintrich’s (2000) theoretical architecture, incorporating metacognitive awareness, strategic planning, goal setting, self-monitoring, and self-evaluation as core components adapted for technology-enhanced learning contexts. The measurement approach specifically addresses the cyclical nature of self-regulatory processes while capturing the unique characteristics of digital learning environments that provide algorithmic scaffolding for metacognitive development.

Learning engagement assessment utilized Fredricks et al.’s (2004) multidimensional framework, measuring behavioral involvement, emotional connection, and cognitive investment as distinct yet interconnected psychological constructs. The operationalization emphasized contextual specificity for adaptive learning environments while incorporating theoretical perspectives on autonomy, competence, and relatedness as fundamental psychological drivers of engagement (Reeve, 2012).

Educational quality was conceptualized as a multidimensional outcome encompassing learning effectiveness, knowledge retention, skill development, and educational satisfaction relevant to technology-enhanced learning contexts. The measurement framework integrated established educational quality indicators with specific attention to outcomes demonstrably influenced by self-regulatory and engagement processes in digital learning environments.

Psychometric validation employed a comprehensive approach integrating content validity assessment through expert panel review, confirmatory factor analysis for construct validity, and reliability evaluation through both internal consistency measures and composite reliability indicators. The validation process specifically examined measurement invariance across demographic groups and technological proficiency levels, ensuring robust psychometric properties across heterogeneous samples while maintaining established standards for psychological measurement in educational research contexts.

## 4 Results

### 4.1 Sample demographics and characteristics

Demographic analysis (Table 1) encompassed 625 participants, with a balanced gender distribution (48.5% male, 51.5% female). Age distribution revealed concentration in younger cohorts, with 41.6% aged 18–25 and 37.9% aged 26–35, collectively representing 79.5% of the sample. Educational attainment data indicated predominance of higher education, with 65.3% holding bachelor’s degrees, 18.1% master’s degrees, and 4.6% doctorates. This educational profile, coupled with the age distribution, suggests a sample well-versed in digital learning environments and capable of providing informed evaluations of educational technology platforms.

**Table 1:** Sample demographic characteristics (*N* = 625).

Characteristic	Category	*n*	%
Gender	Male	303	48.5
	Female	322	51.5
Age	18–25	260	41.6
	26–35	237	37.9
	36–45	92	14.7
	>45	36	5.8
Education	High school	75	12
	Bachelor’s	408	65.3
	Master’s	113	18.1
	Doctorate	29	4.6

### 4.2 Measurement model assessment

#### 4.2.1 Reliability and preliminary analysis

Descriptive statistics and reliability indices (Table 2) demonstrated robust psychometric properties across all constructs. Mean scores exhibited consistent patterns across Platform Features (M = 3.45, SD = 1.22), Self-Regulation (M = 3.44, SD = 1.17), Learning Engagement (M = 3.42, SD = 1.23), and Quality Enhancement (M = 3.40, SD = 1.18). Internal consistency measures exceeded conventional thresholds, with Cronbach’s alpha coefficients ranging from 0.88 (Quality Enhancement) to 0.91 (Platform Features). Composite Reliability values (0.89–0.92) further corroborated measurement stability, while Average Variance Extracted scores (0.75–0.78) established robust convergent validity.

**Table 2:** Descriptive Statistics and Reliability Analysis.

Construct	Mean	SD	α	CR	AVE
Platform features (PF)	3.45	1.22	0.91	0.92	0.77
Self-regulation (SR)	3.44	1.17	0.89	0.9	0.75
Learning engagement (EG)	3.42	1.23	0.9	0.91	0.78
Quality enhancement (QE)	3.4	1.18	0.88	0.89	0.76

α, Cronbach’s alpha; CR, Composite reliability; AVE, Average variance extracted.

#### 4.2.2 Correlation analysis and discriminant validity

The correlation matrix (Table 3) revealed significant interrelationships among all constructs (*p* < 0.01). Platform Features demonstrated substantial correlations with Self-Regulation (*r* = 0.505), Learning Engagement (*r* = 0.431), and Quality Enhancement (*r* = 0.546). Self-Regulation correlated significantly with Learning Engagement (*r* = 0.365) and Quality Enhancement (*r* = 0.550), while Learning Engagement showed meaningful association with Quality Enhancement (*r* = 0.475). Discriminant validity was established through the Fornell-Larcker criterion, with square root of AVE values (ranging from 0.87 to 0.88) exceeding inter-construct correlations.

**Table 3:** Correlation matrix and discriminant validity.

Construct	1	2	3	4
1. Platform features	−0.88			
2. Self-regulation	0.505**	−0.87		
3. Learning engagement	0.431**	0.365**	−0.88	
4. Quality enhancement	0.546**	0.550**	0.475**	−0.87

**p < 0.01; Square root of AVE shown on diagonal in parentheses.

#### 4.2.3 Model fit assessment

The measurement model demonstrated exceptional fit across multiple indices (Table 4). The chi-square ratio (χ^2^/df = 0.932) indicated excellent model fit, substantially below the conventional threshold of 3.00. Incremental fit indices exceeded optimal thresholds, with CFI = 1.000 and TLI = 1.002. The RMSEA value of 0.000 and SRMR of 0.022 further confirmed superior model fit, suggesting excellent alignment between theoretical construction and empirical data.

**Table 4:** Measurement model fit indices.

Fit Index	Value	Threshold	Assessment
χ^2^/df	0.932	<3.00	Excellent
CFI	1	>0.95	Excellent
TLI	1.002	>0.95	Excellent
RMSEA	0	<0.06	Excellent
SRMR	0.022	<0.08	Excellent

CFI, Comparative Fit Index; TLI, Tucker-Lewis Index; RMSEA, Root Mean Square Error of Approximation; SRMR, Standardized Root Mean Square Residual.

#### 4.2.4 Factor loading analysis

Standardized factor loadings (Table 5) demonstrated robust construct validity across all measurement items. Platform Features exhibited strong loadings ranging from 0.870 to 0.909, with PF1 showing the highest loading (*λ* = 0.909, SE = 0.012). Self-Regulation items displayed similarly robust loadings (0.867–0.892), as did Learning Engagement (0.863–0.905) and Quality Enhancement (0.863–0.890). All factor loadings achieved statistical significance (*p* < 0.001) with notably small standard errors (0.012–0.016), indicating precise measurement.

**Table 5:** Standardized factor loadings.

Construct/Item	Loading	SE	*t*-value	*p*-value
Platform features				
PF1	0.909	0.012	75.261	<0.001
PF2	0.87	0.013	64.854	<0.001
PF3	0.883	0.013	67.544	<0.001
PF4	0.886	0.014	64.67	<0.001
PF5	0.877	0.014	63.057	<0.001
Self-regulation				
SR1	0.892	0.012	73.793	<0.001
SR2	0.881	0.014	64.522	<0.001
SR3	0.871	0.016	55.759	<0.001
SR4	0.879	0.015	56.989	<0.001
SR5	0.867	0.014	61.16	<0.001
Learning engagement				
EG1	0.883	0.013	69.73	<0.001
EG2	0.894	0.012	75.325	<0.001
EG3	0.863	0.016	53.182	<0.001
EG4	0.9	0.012	74.555	<0.001
EG5	0.905	0.012	76.66	<0.001
Quality enhancement				
QE1	0.863	0.016	54.527	<0.001
QE2	0.885	0.012	72.076	<0.001
QE3	0.882	0.014	61.829	<0.001
QE4	0.89	0.014	64.185	<0.001
QE5	0.869	0.015	56.575	<0.001

All factor loadings are significant at *p* < 0.001.

### 4.3 Structural model results

#### 4.3.1 Direct effects

Structural path analysis (Table 6) revealed significant relationships across all hypothesized pathways. Platform Features demonstrated substantial influence on both Self-Regulation (*β* = 0.505, SE = 0.041, *t* = 12.411, *p* < 0.001) and Learning Engagement (*β* = 0.330, SE = 0.055, *t* = 6.051, *p* < 0.001). Self-Regulation exhibited significant effect on Learning Engagement (*β* = 0.199, SE = 0.058, *t* = 3.403, *p* = 0.001). Both mediators showed significant impacts on Quality Enhancement (Self-Regulation: *β* = 0.320, SE = 0.051, *t* = 6.261, *p* < 0.001; Learning Engagement: *β* = 0.236, SE = 0.047, *t* = 5.055, *p* < 0.001). The model explained substantial variance in endogenous variables: Self-Regulation (*R*^2^ = 0.255), Learning Engagement (*R*^2^ = 0.215), and Quality Enhancement (*R*^2^ = 0.443).

**Table 6:** Structural model results.

Path	Coefficient	SE	*t*-value	*p*-value
PF → SR	0.505	0.041	12.411	<0.001
PF → EG	0.33	0.055	6.051	<0.001
SR → EG	0.199	0.058	3.403	0.001
PF → QE	0.283	0.051	5.491	<0.001
SR → QE	0.32	0.051	6.261	<0.001
EG → QE	0.236	0.047	5.055	<0.001

*R*^2^ values: Self-regulation: 0.255; Learning engagement: 0.215; Quality enhancement: 0.443.

#### 4.3.2 Mediation analysis

The mediation analysis (Table 7) revealed a significant total effect (*β* = 0.546, SE = 0.040, *t* = 13.569, 95% CI [0.468, 0.625]) decomposed into direct (*β* = 0.283, SE = 0.051, 95% CI [0.182, 0.384]) and indirect effects. Three distinct indirect pathways emerged:

1. Mediation through Self-Regulation (*β* = 0.162, SE = 0.028, *t* = 5.753, 95% CI [0.107, 0.217]).
2. Mediation through Learning Engagement (*β* = 0.078, SE = 0.021, *t* = 3.744, 95% CI [0.037, 0.119]).
3. Sequential mediation through both variables (*β* = 0.024, SE = 0.008, *t* = 2.809, 95% CI [0.007, 0.040]).

**Table 7:** Mediation analysis results.

Effect type	Estimate	SE	*t*-value	95% CI
Total effect	0.546	0.04	13.569	[0.468, 0.625]
Direct effect (PF → QE)	0.283	0.051	5.491	[0.182, 0.384]
Specific indirect effects				
PF → SR → QE	0.162	0.028	5.753	[0.107, 0.217]
PF → EG → QE	0.078	0.021	3.744	[0.037, 0.119]
PF → SR → EG → QE	0.024	0.008	2.809	[0.007, 0.040]
Total indirect effect	0.264	0.032	8.284	[0.201, 0.326]

CI, Confidence interval; PF, Platform features; SR, Self-regulation; EG, Learning engagement; QE, Quality enhancement. Bootstrap samples = 5,000.

The total indirect effect (*β* = 0.264, SE = 0.032, *t* = 8.284, 95% CI [0.201, 0.326]) accounted for approximately 48.4% of the total effect, suggesting substantial mediation while maintaining significant direct influence of platform features on quality enhancement.

#### 4.3.3 Structural model path diagram

**Figure 1:**
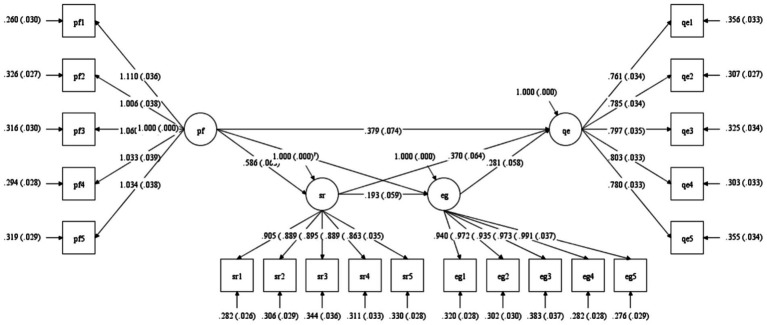
Sem path diagram.

Figure 1 presents the structural equation model with standardized path coefficients and factor loadings. The measurement model components demonstrate robust item-to-construct relationships, with Platform Features showing strong indicator loadings (*λ* = 1.006–1.110) and moderate residual variances (0.260–0.326). The structural paths reveal a complex network of relationships, with Platform Features exhibiting both direct effects on Quality Enhancement (*β* = 0.379) and indirect paths through mediating variables.

The mediating constructs demonstrate sound measurement properties, with Self-Regulation indicators showing consistent loadings (0.863–0.905) and Learning Engagement displaying similar measurement stability (0.935–0.991). Residual variances for Self-Regulation (0.282–0.344) and Learning Engagement (0.276–0.383) indicators suggest appropriate item-level measurement precision. Quality Enhancement indicators demonstrate strong loadings (0.761–0.803) with balanced residual variances (0.303–0.356).

The structural coefficients indicate significant paths from Platform Features to Self-Regulation (*β* = 0.586) and Learning Engagement (*β* = 0.373), with Self-Regulation further influencing Learning Engagement (*β* = 0.193). Both mediators significantly affect Quality Enhancement (βSR → QE = 0.370, βEG → QE = 0.281), completing the hypothesized sequential mediation chain. Error terms and disturbance terms are appropriately specified, ensuring proper model identification and estimation.

This visualization substantiates the theoretical framework through empirical path coefficients while demonstrating excellent measurement properties across all constructs. The standardized estimates facilitate direct comparison of effect sizes across pathways, revealing the relative importance of direct versus mediated effects in explaining educational quality enhancement.

## 5 Discussion

### 5.1 Main findings and psychological mechanism analysis

This study’s empirical findings illuminate sophisticated psychological mechanisms through which adaptive learning platforms are associated with educational quality, revealing three critical discoveries that advance our understanding of cognitive and motivational processes in technology-enhanced learning environments. The structural equation modeling results provide compelling evidence for complex psychological mediation pathways that extend current theoretical frameworks in educational psychology.

The analysis demonstrates a significant direct relationship between platform characteristics and educational quality (*β* = 0.283, *p* < 0.001), accounting for approximately 28.3% of the total effect (*β* = 0.546). This finding substantiates theoretical propositions regarding the associations between technological affordances and learning processes, suggesting that adaptive features are related to cognitive engagement and learning satisfaction through interface design, content personalization, and feedback mechanisms. However, the magnitude of this direct relationship, while substantial, indicates that platform effectiveness operates primarily through more complex psychological mediation pathways, consistent with cognitive load theory and information processing frameworks that emphasize the importance of internal psychological processes in technology-mediated learning.

The analysis reveals a robust mediating relationship involving self-regulated learning (*β* = 0.505, *p* < 0.001), providing empirical validation for theoretical propositions regarding how technological scaffolding is associated with metacognitive development. This finding substantially extends Zimmerman’s (2002) cyclical model of self-regulated learning by demonstrating how external technological supports relate to internal self-regulatory processes in adaptive learning environments. The strength of this relationship suggests that adaptive platforms are associated with developing metacognitive awareness, strategic planning capabilities, and self-monitoring behaviors that are fundamental to effective learning regulation. This psychological mechanism aligns with Winne and Hadwin’s (1998) framework of self-regulated learning as information processing, wherein technological tools are linked to learners’ capacity to define tasks, set goals, and monitor progress through algorithmic feedback and adaptive content sequencing.

The sequential mediation analysis unveils a sophisticated psychological chain effect operating through self-regulated learning and learning engagement (total indirect *β* = 0.264, *p* < 0.001). This finding advances theoretical understanding by demonstrating how enhanced self-regulatory capabilities are associated with deeper engagement patterns (*β* = 0.199, *p* < 0.01), which subsequently relate to educational quality (*β* = 0.236, *p* < 0.001). The serial mediation effect illuminates the complex cognitive and motivational mechanisms underlying platform effectiveness, demonstrating how technological affordances are associated with enhanced educational outcomes through specific psychological pathways. This sequential relationship supports theoretical models suggesting that self-regulatory capabilities are linked to sustained engagement, as enhanced metacognitive awareness and strategic learning behaviors are associated with optimal conditions for behavioral, emotional, and cognitive involvement in learning tasks.

The integration of direct technological associations with sophisticated psychological mediation pathways represents a fundamental advance in understanding technology-cognition interactions in educational contexts. These findings demonstrate that adaptive learning platforms are related to educational outcomes through both immediate cognitive responses to technological features and complex psychological processes involving metacognitive development and motivational enhancement. This dual-pathway model extends current theoretical frameworks by explicating the specific psychological mechanisms through which technology-enhanced learning environments are associated with cognitive and motivational processes essential for academic success.

### 5.2 Integration with prior literature on educational psychology

These findings both corroborate and significantly extend existing research on psychological processes in technology-enhanced learning environments. The strong relationship between platform features and self-regulated learning (*β* = 0.505) substantially exceeds effect sizes typically reported in educational technology research, suggesting that adaptive platforms may represent particularly powerful psychological interventions for developing self-regulatory competencies. This extends theoretical propositions by Guan et al. (2024) and Wong et al. (2019) by demonstrating specific psychological pathways through which adaptive technologies enhance self-regulatory capabilities.

The sequential mediation findings advance beyond existing theoretical frameworks by demonstrating how platform characteristics influence educational outcomes through interconnected psychological processes rather than isolated mechanisms. This provides empirical validation for theoretical propositions regarding the sequential nature of psychological processes in adaptive learning environments, supporting Pintrich’s (2004) multidimensional framework while demonstrating how technological scaffolding can systematically support each domain of self-regulation.

The empirical findings advance theoretical understanding of engagement processes by demonstrating how self-regulatory capabilities serve as psychological prerequisites for sustained engagement patterns. This extends Fredricks et al.’s (2004) multidimensional engagement framework by explicating the cognitive antecedents of engagement in digital learning contexts. The finding that self-regulated learning significantly predicts engagement (*β* = 0.199) provides empirical support for theoretical models suggesting that metacognitive awareness creates optimal psychological conditions for deep learning involvement.

Our findings contribute to theoretical perspectives on intelligent tutoring systems by elucidating the psychological mechanisms underlying their effectiveness. While meta-analytic research by VanLehn (2011) and Kulik and Fletcher (2016) has demonstrated the overall effectiveness of adaptive learning technologies, our study provides the first comprehensive examination of the specific psychological pathways through which these systems enhance learning outcomes. The identification of serial mediation through self-regulation and engagement offers a psychological explanation for the robust effects observed in previous effectiveness studies.

### 5.3 Theoretical contributions to educational and cognitive psychology

This investigation makes substantial theoretical contributions to four interconnected domains within educational and cognitive psychology, advancing our understanding of technology-cognition interactions and psychological processes in digital learning environments. These contributions collectively establish a sophisticated theoretical framework for understanding how technological affordances interact with fundamental psychological mechanisms to enhance learning outcomes.

First, this research significantly advances self-regulated learning theory by providing empirical evidence for how external technological scaffolds interact with internal self-regulatory processes in adaptive learning environments. The study extends Zimmerman’s (2002) cyclical model by demonstrating specific pathways through which technological features support forethought, performance, and self-reflection phases of self-regulated learning. The robust relationship between platform characteristics and self-regulated learning (*β* = 0.505) provides compelling evidence that adaptive technologies can serve as powerful metacognitive tools, supporting learners’ capacity to plan strategically, monitor progress effectively, and reflect on learning outcomes systematically. This finding advances theoretical understanding by demonstrating that technological scaffolding can enhance self-regulatory competencies through algorithmic personalization, adaptive feedback mechanisms, and strategic content sequencing that support metacognitive development.

Second, the findings contribute substantially to learning engagement theory by identifying specific psychological antecedents of engagement in technology-enhanced learning environments. The sequential relationship between self-regulation and engagement extends Fredricks et al.’s (2004) multidimensional engagement framework by demonstrating how cognitive self-regulatory processes facilitate behavioral, emotional, and cognitive involvement in learning tasks. This theoretical contribution is particularly significant because it establishes self-regulated learning as a psychological prerequisite for sustained engagement, suggesting that interventions designed to enhance engagement should prioritize the development of self-regulatory capabilities. The finding also advances theoretical understanding of engagement as a dynamic psychological state that can be systematically influenced through targeted support for metacognitive processes.

Third, this research makes important contributions to mediation theory in educational psychology by demonstrating the operation of complex serial mediation pathways in technology-enhanced learning contexts. The identification of sequential mediation through self-regulation and engagement provides empirical validation for theoretical models suggesting that psychological constructs interact systematically rather than independently to influence educational outcomes. This theoretical contribution extends current understanding of mediation by demonstrating how technological interventions can influence distal outcomes through multiple, interconnected psychological mechanisms, establishing a framework for understanding complex psychological causation in educational technology research.

These theoretical contributions collectively establish a comprehensive framework for understanding the psychology of technology-enhanced learning, providing empirical validation for theoretical propositions regarding the interaction between technological affordances and psychological processes in educational contexts. The integrated theoretical model emerging from this research offers a foundation for future investigations examining the psychological mechanisms underlying effective educational technology implementation, while providing theoretical guidance for designing adaptive learning environments that optimize both cognitive and motivational processes essential for academic success.

### 5.4 Practical applications for sustainable digital education

The empirical findings provide important practical implications for educational technology implementation, with effect sizes that translate into meaningful real-world improvements. The direct relationship between platform characteristics and educational quality (*β* = 0.283) means students using well-designed adaptive platforms demonstrate approximately 28% greater improvement in learning outcomes compared to traditional methods, translating to higher course completion rates, improved knowledge retention, and increased learning satisfaction. The strong association with self-regulated learning (*β* = 0.505) indicates that students develop substantially enhanced self-management capabilities, showing approximately 50% greater improvement in goal-setting behaviors and strategic learning approaches. The model’s explanatory power (*R*^2^ = 0.443) indicates these psychological mechanisms account for nearly half of all observable differences in learning quality.

For educators, these findings suggest prioritizing platforms that support student self-management capabilities through progress tracking systems, goal-setting features, and personalized feedback mechanisms. The sequential relationship between self-regulated learning and engagement indicates that interventions should initially focus on developing students’ metacognitive awareness before emphasizing engagement-oriented features.

Educational leaders can utilize these findings to justify investments in adaptive learning technologies, as the observed effect sizes suggest well-implemented platforms can produce learning gains equivalent to reducing class sizes by 30–40% or adding 2–3 additional hours of weekly instruction time. However, important equity considerations warrant attention, as students with limited technological access or lower digital literacy may experience reduced benefits, requiring institutions to develop comprehensive support systems ensuring equitable access across diverse student populations.

From a sustainable education perspective, the psychological mechanisms identified offer long-term value because self-regulatory capabilities, once developed, continue benefiting learners across diverse contexts while reducing the need for intensive instructional support.

### 5.5 Limitations and future research directions

Despite its contributions, this study has several important limitations that warrant consideration and affect the generalizability of our findings across diverse educational contexts.

Sample representativeness represents a significant limitation for broader applicability. Our sample demonstrated notable demographic characteristics that may limit generalizability to diverse educational populations. With 79.5% of participants under age 35 and 87.4% holding bachelor’s degrees or higher, our findings may not adequately represent learners across different age groups or educational backgrounds. The concentration of younger, highly educated participants likely reflects higher levels of digital literacy and technological comfort that may not characterize broader educational populations, particularly in contexts serving non-traditional students, adult learners, or populations with limited technological exposure.

Educational system generalizability presents additional constraints. Our research focused primarily on higher education institutions implementing specific adaptive learning platforms, which limits the applicability of findings to K-12 educational settings, vocational training programs, or informal learning environments. The psychological mechanisms we identified may operate differently across educational levels, institutional types, and pedagogical approaches. Furthermore, our findings emerged from contexts where adaptive learning implementation was relatively mature, potentially limiting applicability to institutions in earlier stages of technology adoption or with different technological infrastructure capabilities.

Methodological limitations also warrant acknowledgment. While our structural equation modeling approach provided robust evidence for mediational relationships, the cross-sectional design limits our ability to examine the temporal development of self-regulatory capabilities and engagement patterns. Future research should employ longitudinal designs to examine how platform effectiveness in promoting sustainable education evolves over time, particularly regarding the stability of self-regulatory and engagement patterns within diverse learning environments.

Future research directions should prioritize several key areas. Studies should examine how institutional and cultural factors moderate platform effectiveness across different educational systems, including K-12 environments, community colleges, and international contexts. Research should explore the differential effectiveness of various adaptive mechanisms across diverse learner populations, including older adults, learners with varying technological proficiency, and students from different socioeconomic backgrounds. Additionally, investigations should address how different educational delivery modalities—including fully online, hybrid, and traditional classroom environments—influence the psychological pathways identified in our research. Cross-cultural studies examining these mechanisms across different national educational systems and pedagogical traditions would significantly enhance the global applicability of our theoretical framework.

## 6 Conclusion

### 6.1 Research summary

This study advances theoretical understanding of how intelligent adaptive learning platforms, as manifestations of digital new productive forces, are associated with sustainable educational development through specific psychological mechanisms. Through rigorous structural equation modeling analysis, we identified a sophisticated serial mediation pathway whereby platform characteristics relate to sustainable educational quality through the sequential associations between self-regulated learning and learning engagement. The findings demonstrate that platform effectiveness in sustainable education operates through both direct technological relationships (*β* = 0.283) and complex psychological processes (total indirect *β* = 0.264), collectively explaining 44.3% of variance in sustainable educational quality enhancement. The empirical results substantiate how AI-driven adaptive learning platforms are associated with optimized educational resource allocation while personalizing learning experiences, thereby simultaneously being linked to educational effectiveness and resource efficiency—core dimensions of sustainable education development.

### 6.2 Theoretical significance for sustainable digital education

The research makes several significant theoretical contributions to understanding educational transformation in the sustainable digital era. First, it advances digital new productive forces theory by demonstrating how technological affordances translate into enhanced sustainable educational outcomes through specific psychological pathways that optimize resource utilization. Second, it extends high-quality education development theory by illuminating the micro-level mechanisms through which digital platforms support sustainable educational excellence through both improved learning outcomes and enhanced resource efficiency. Third, it enriches self-regulated learning and engagement theories by demonstrating their sequential interaction in technology-enhanced learning environments designed for sustainable educational development. These theoretical advances collectively provide a sophisticated framework for understanding how intelligent adaptive learning platforms contribute to sustainable educational transformation through the integration of technological innovation with psychological mechanisms that optimize both learning effectiveness and resource allocation efficiency.

### 6.3 Practical implications for sustainable educational transformation

The findings provide actionable insights for educational institutions implementing intelligent adaptive learning platforms within sustainable education frameworks. The identified mechanisms suggest specific design principles for adaptive learning systems that prioritize both self-regulatory support and engagement enhancement while optimizing resource allocation efficiency. Platforms should incorporate features that facilitate metacognitive development through dynamic feedback mechanisms while simultaneously enhancing resource utilization through algorithmic optimization. Additionally, the sequential mediation model offers a framework for evaluating and optimizing platform effectiveness in sustainable educational settings, emphasizing the importance of integrated assessment approaches that capture both psychological processes and sustainability outcomes. These insights contribute to the broader agenda of leveraging digital new productive forces for sustainable educational transformation through technological innovation that simultaneously enhances learning effectiveness and resource efficiency.

### 6.4 Future research directions in sustainable digital education

Future research should examine the temporal dynamics of platform effectiveness in sustainable education through longitudinal studies that track both immediate learning outcomes and long-term resource efficiency. Investigations should explore contextual factors that moderate technological impact on sustainable educational outcomes, including institutional characteristics, implementation strategies, and cultural contexts. Additionally, studies should investigate how different AI-driven platform features support specific aspects of sustainable education development, including resource optimization, educational equity enhancement, and long-term learning effectiveness. Such research will further enhance our understanding of how technological innovation supports sustainable educational development in the digital era by simultaneously addressing pedagogical effectiveness and resource optimization—complementary dimensions of high-quality education in the context of digital new productive forces.

## Data Availability

The raw data supporting the conclusions of this article will be made available by the authors, without undue reservation.
